# A Review on Konjac Glucomannan Gels: Microstructure and Application

**DOI:** 10.3390/ijms18112250

**Published:** 2017-10-27

**Authors:** Dan Yang, Yi Yuan, Lin Wang, Xiaoshan Wang, Ruojun Mu, Jie Pang, Jianbo Xiao, Yafeng Zheng

**Affiliations:** 1 College of Food Science, Fujian Agriculture and Forestry University, Fuzhou 350002, China; yangd1728@163.com (D.Y.); yuanjohnay1233@163.com (Y.Y.); brandonwjl53667@163.com (L.W.); muruojun@gmail.com (R.M.); 2 College of Materials and Engineering, Fujian Agriculture and Forestry University, Fuzhou 350002, China; bombomhoo@163.com; 3 Institute of Chinese Medical Sciences, State Key Laboratory of Quality Control in Chinese Medicine, University of Macau, Macau 999078, China; 4 Fujian Provincial Key Laboratory of Quality Science and Processing Technology in Special Starch, Fujian Agriculture and Forestry University, Fuzhou 350002, China

**Keywords:** konjac glucomannan gel, preparation, microstructure, application

## Abstract

Konjac glucomannan (KGM) has attracted extensive attention because of its biodegradable, non-toxic, harmless, and biocompatible features. Its gelation performance is one of its most significant characteristics and enables wide applications of KGM gels in food, chemical, pharmaceutical, materials, and other fields. Herein, different preparation methods of KGM gels and their microstructures were reviewed. In addition, KGM applications have been theoretically modeled for future uses.

## 1. Introduction

Konjac glucomannan (KGM) is a water-soluble polysaccharide separated from konjac tubers. KGM is a dietary fiber with a long history in food and traditional Chinese medicine [1,2,3]. The KGM main chain is polymerized by d-mannose and d-glucose with a α-1,4-pyranoside bond and a small amount of acetyl groups at the C–6 position of the side chain. These can only be hydrolyzed by α-mannase at the end of the small intestine and the colon of the human body [4,5,6]. KGM has good film-forming ability, biocompatibility, biodegradability, and gelation performance, which is one of its most prominent features [2,7,8]. The preparation methods of KGM gel mainly include the alkaline processing [9,10], borate cross-linking [11,12], polymer compounding [13,14], high voltage electric field preparation [15,16], and metal ion cross-linking after modification [17,18]. The gel microstructure largely determines the performance of the gel, but KGM gels prepared through different methods are significantly different in terms of microstructure [19,20,21]. With good biocompatibility and biodegradability [22,23,24], KGM gels have been widely used in food [19,21,25], pharmaceutical carriers [14,26], tissue scaffolds [27,28], absorbing materials [2,29] and other fields. In this study, different KGM gel preparation methods and the gel microstructures were reviewed. In addition, the application of KGM gel was also discussed to provide new directions for further studies on KGM gel.

## 2. Preparation of Konjac Glucomannan (KGM) Gel and Its Microstructure

### 2.1. Alkaline Processing

Extensive studies have shown that the acetyl groups in the KGM molecular chain are a determining factor for some properties of KGM, especially for its gelation property [8]. Original KGM cannot prepare gel, alkaline processing is the most widely used method to prepare KGM gels. Deacetylated KGM (Da-KGM) is obtained by alkaline processing KGM, can be used to prepare thermally irreversible gels. As the degree of deacetylation increases, the gelation velocity will increase along with improved elastic modulus. This is because deacetylation causes the KGM molecular chain to change from semi-crimping to self-crimping. This leads to self-aggregation between the KGM molecules [9,10,30]. Generally, for the preparation of KGM gels, Da-KGM sols need to be heated for the polymerization of KGM molecules. However, for the further improving the properties of KGM gels, other post-processing (freeze-thawing, freeze drying, etc.) and additives (graphene oxide, sodium montmorillonite, carbon nano-tube, etc.) are used. In addition, Da-KGM also can be used as the component to enhance the properties of other gels.

**Figure 1:**
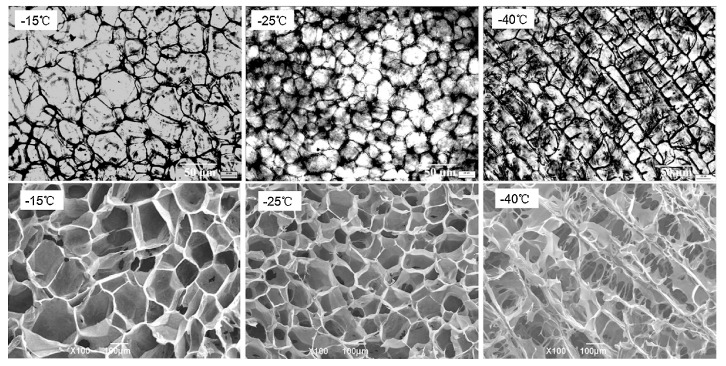
Microstructure of ice crystals (1st row, low temperature polarizing microscopy) and aerogels (2nd row, scanning electron microscopy) formed under different pre-freezing temperatures (−15, −25, and −40 °C) [31].

As an effective method to prepare hydrogels, freeze-thawing method has been widely used in the preparation of Da-KGM-based hydrogels. We also know that frozen hydrogels can be used to prepared aerogels by freeze-drying. Freeze is the most important procedure for freeze-thawing and freeze-drying methods, this is due to the network of gels will be formed in this procedure, and the ice crystal grow under freezing can control the porous structure of network [31]. As shown in Figure 1, pre-freezing temperature has a great influence on the formation of ice crystals. In summary, the lower temperature, the smaller ice crystals and the more uniform spherical shape. Moreover, the scanning electron microscope (SEM) images in Figure 1 shown that the pore size of aerogels also closely dependent on pre-freezing temperature, this is due to the vacuum sublimation of ice crystals [31]. Due to the weak properties, pure Da-KGM hydrogel prepared by freeze-thawing method cannot meet actual applications, therefore, some additives are used in the procedure. graphene oxide (GO) is a common additive for enhancing properties of materials, Yuan et al. [32] successfully prepared KGM/GO hydrogels by alkaline processing followed freeze-thawing, KGM/GO hydrogel showed a compact structure and decreased pore size comparing with pure KGM hydrogel. The porous structures of KGM/GO hydrogel had great specific surface area, and provided a possibility for drug storage and sustained delivery. Moreover, this structure also showed the interaction between KGM and GO, and it was agreement with other characterizations.

At the same time, Da-KGM also can be used to prepare KGM-based aerogels by freeze-drying method. A series of Da-KGM-based aerogels were prepared by Ye et al. [33,34,35,36], and showed well absorption effects for dyes and arsenic. Magnetic Fe and Mn oxides (Mag-FMBO) containing sodium montmorillonite (Na^+^-MMT)-reinforced Da-KGM-based aerogels were fabricated through sol-gel process followed by freeze-drying, Na^+^-MMT and Mag-FMBO were used as the functional additives in that study. All the composite aerogels exhibited abundant porous structure, but the higher inclusion of Mag-FMBO will lead the rougher surfaces [33]. KGM-based magnetic carbon aerogels were successfully fabricated through carbonizing above Mag-FMBO containing Na^+^-MMT-reinforced KGM-based aerogels, and showed an excellent adsorption performance towards anionic methyl orange (MO) and cationic methylene blue (MB), the maximum MO and MB uptake capacity of the aerogels reached 7.42 mg/g and 9.37 mg/g, respectively [34]. Da-KGM/GO/FMBO composite aerogels was fabricated by adding GO on the preparation of Mag-FMBO containing Na^+^-MMT-reinforced KGM-based aerogels. All the composite aerogels samples exhibited abundant porous structure, but the content of GO showed significant effect on the composite aerogels’ surfaces [35].

**Figure 2:**
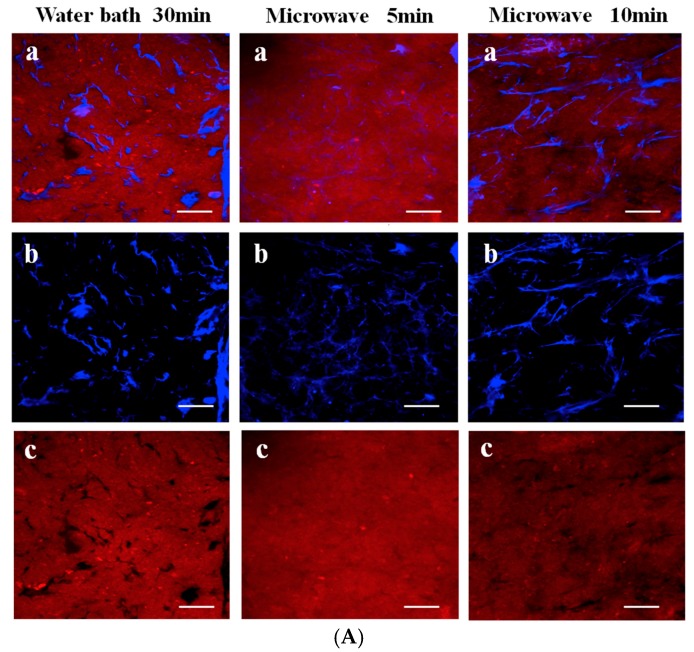

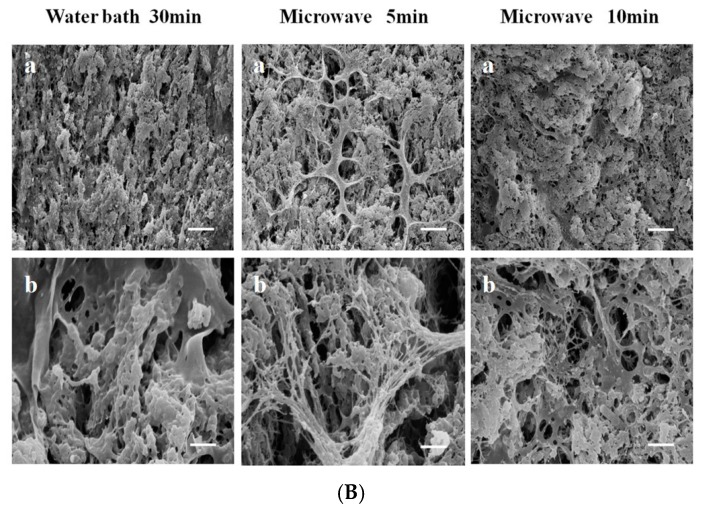
(**A**). CLSM images of glucomannan-protein mixed gels at room temperature (25 °C) after treated with different heating methods. (**a**) Microstructure (labeled protein and glucomannan) of glucomannan-protein mixed gels; (**b**) Microstructures (labeled glucomannan) of glucomannan-protein mixed gels; (**c**) Microstructures (labeled protein) of glucomannan-protein mixed gels. The scale bar indicates 20 μm; (**B**). SEM micrographs (1000× and 5000×) for glucomannan-protein mixed gels treated with different heating methods. (**a**) Microstructures (1000×) of glucomannan-protein mixed gels, the scale bar indicates 10 μm; (**b**) Microstructures (5000×) of glucomannan-protein mixed gels, the scale bar indicates 2 μm [19].

In addition to using Da-KGM to prepare KGM-based gels though freeze-thawing (or freeze-drying) method, Da-KGM-polymer complexes hydrogels also can be prepared. Wheat starch gels were successfully produced with KGM and low concentrations of Na_2_CO_3_ by Zhou et al. [37], confocal laser scanning microscopy (CLSM) was used to study the morphological structure of starch gels. According to the results, Na_2_CO_3_ promoted the formation of fiber-like extensions around scattered swollen starch granules by KGM and amylose interaction, and led the phase dispersion of KGM-starch gels. Ji et al. [19] studied the impact of the two heating methods on the structure and quality of KGM-Alaska pollock surimi protein composite gels and used the CLSM and SEM to measure the microstructure, elongation, and dispersion of KGM in protein chains as well as the microstructure of the mixed gel. The microstructure and interaction of KGM and protein molecular chains were observed by drying KGM and protein differently. The CLSM in Figure 2A shows that the gel prepared by microwave heating has a high elongation rate. The KGM and protein formed a good network structure, and the KGM network structure was drawn into filaments. However, when water bath heating was applied, the KGM mostly existed in the protein molecular chain in a coiled state. Different heating methods significantly affect the structure of the KGM-protein gels. The SEM in Figure 2B confirms that microwave heating can produce a more swollen network structure with distinct gullies and a robust skeleton. KGM-Alaska pollock surimi protein composite gels were also prepared though high-temperature treatment (120 °C) by Zhang et al. [38]. The SEM images of gels showed that all of them had a network structure, but the composite gels contained denser and more uniform network structure than pure surimi protein gel. Specially, the composite gels with higher deacetylation of KGM would led more compact network frames and smaller holes in the network.

### 2.2. Borate Cross-Linking

Gao et al. [11] prepared a series of organic borate cross-linked thermally irreversible KGM gels. The gel network was formed by the cross-linking reaction between the borate ion dissociated by the organic borate and the *cis*-diol hydroxyl group on the mannose unit of the polysaccharide chain. The rheological properties of the composite gel were studied by dynamic viscoelasticity measurements. The gelation kinetics of the gel was studied, and the critical gelation point of the gel was accurately determined by the Winter-Chambon standard. This group studied how temperature and the ratio of composite materials affect shear storage modulus (G′), loss modulus (G′′), and sol-gel transition point. The Winter-Chambon standard accurately explained the critical gel-sol temperature of the composite gel.

### 2.3. Polymer Compounding

#### 2.3.1. KGM-Polysaccharide Compounding

Synergistic reaction between KGM and other polysaccharide is another method for preparing KGM-based hydrogels, KGM-κ-carrageenan and KGM-xanthan gum (XG) hydrogels had been reported in previous researches, but their microstructure had not reported [39,40,41,42]. Zhang et al. [43] used two natural polysaccharides—KGM and XG—to prepare a mixed gel film for use as a matrix material for mucosal adhesives and transdermal drug delivery systems. Estimation and experimental verification suggested that the optimum formulation was as follows: 50 mL of 1% (*w*/*w*) KGM and 1% (*w*/*w*) XG solution were used to prepare the mixed gel at a ratio of 2.36:1 (*v*/*v*). The 0.35 mL glycerol and 0.47 mL tween-80 were then added. The resulting gel film had an optimal adhesive capacity with an adhesive force of (50.85 ± 3.57) g (*n* = 6) for each piece of gel film. The release of acyclovir from the drug membrane reached a maximum after 4.5 h, which could be maintained for 3–7 h. The mixed gel prepared by KGM and XG had a stable network microstructure, good bio-adhesive capacity, and controlled release ability. The results showed that the microstructure of the matrix gel enables its use in mucosal adhesives and transdermal drug delivery systems.

**Figure 3:**
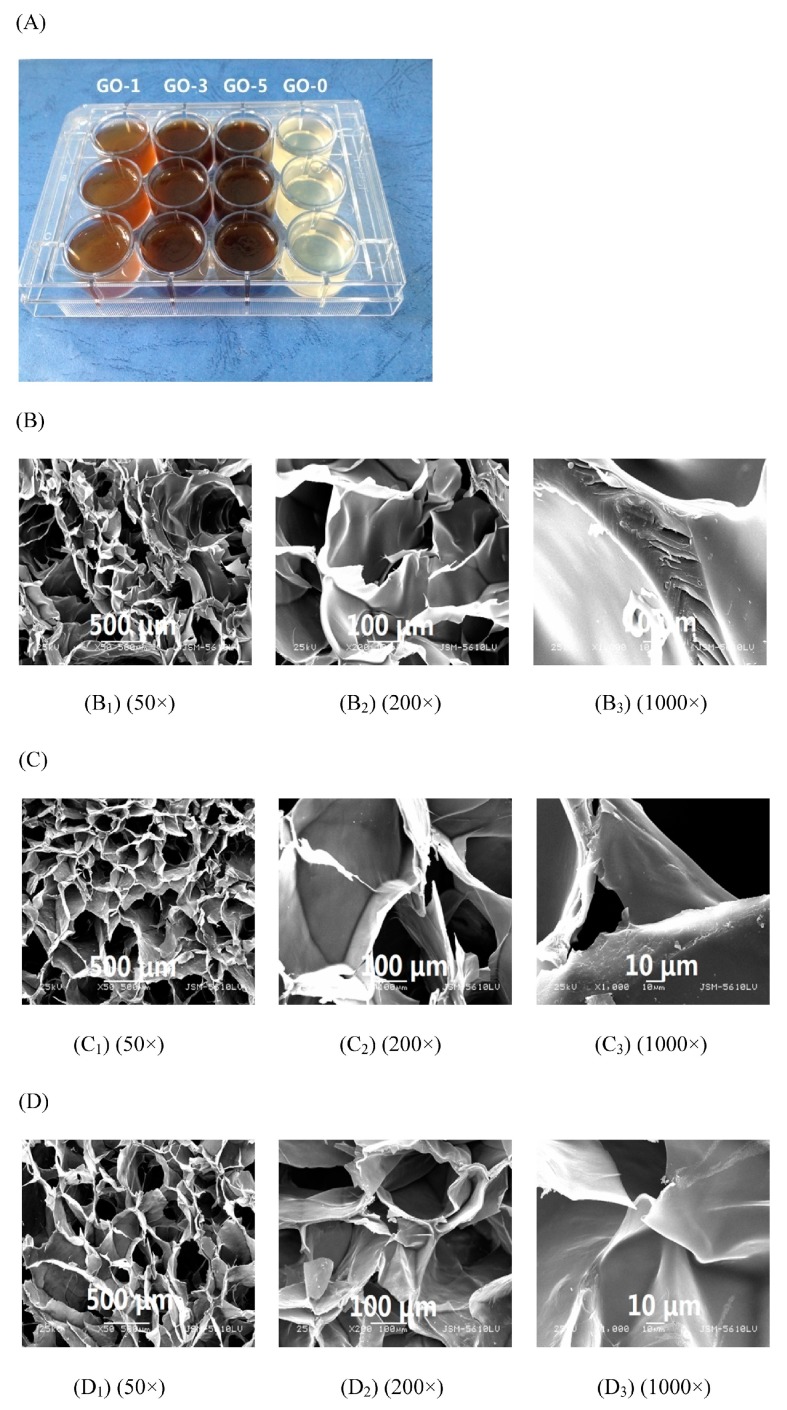
Digital photo of composite hydrogels with different GO loadings. (**A**) SEM images of the cross-section of the GO-0 hydrogel; (**B**) GO-3 hydrogel; (**C**) and GO-5 hydrogel; (**D**) at different magnifications [14].

Polysaccharides’ derivatives are another types of idea materials for the preparation of hydrogels, due to their function groups can be easily interacted for the formation of network, Fan et al. [14] reported that the porous microstructure of the scaffolds has a significant influence on cell invasion, proliferation, and tissue engineering functions. The microstructure of the hydrogel was characterized by SEM and showed that all hydrogel samples have a continuous and stable three-dimensional network structure similar to some polysaccharides and GO hydrogels reported earlier. In addition, all hydrogel samples showed a number of micropores evenly distributed within the hydrogels. The interconnection between the micropores can be attributed to the network formed by the cross-linking between carboxymethyl chitosan (CMCS) and OKGM (KGM oxidized by sodium periodate) and the hydrogen bond between GO nanosheets and polymer chains. When GO increased from 0 to 5 mg/mL there was no significant change in the mean pore diameter of the gel (GO-0 (Figure 3(B_1_)), GO-3 (Figure 3(C_1_)), and GO-5 (Figure 3(D_1_))). The GO-0 sample was torn during the freeze-drying process, and the hydrogel containing GO completely avoided structural collapse during dehydration. The results are consistent with the mechanical properties. The main reason is the strong hydrogen bond interactions between GO and the polymer chain. The GO-containing hydrogel has a more stable network structure compared with the hydrogel without GO.

**Figure 4:**
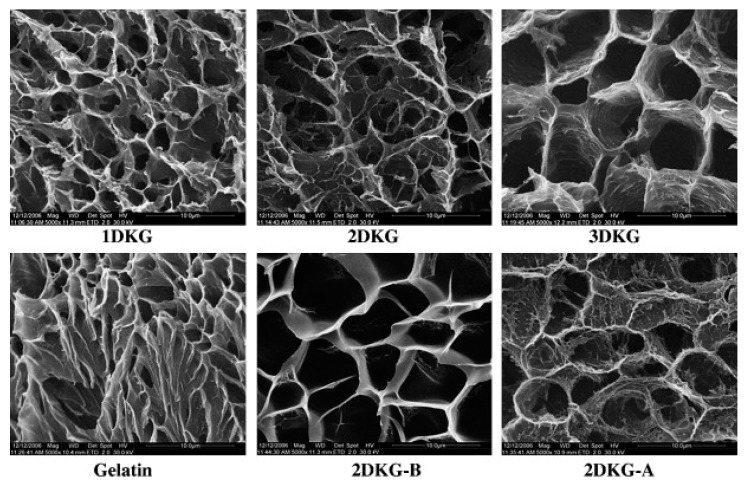
The microstructure of the freeze-dried oxidized KGM (DKG) composite gels, gelatin, and swollen state of 2DKG at pH 9.0 (2DKG-B) and at pH 4.0 (2DKG-A), the scale bar indicates 100 μm [44].

Yu et al. [44] studied oxidized KGM (denoted as DAK) as macromolecule cross-linking agents in the preparation of medicine-carrying hydrogels based on gelatin (GL). The results show that DAK promotes the formation of a gelatin network. More interestingly, the gelatin hydrogel processed with DAK significantly slows the release of the model drug ketoprofen and the release rate can be controlled by the DAK/GL ratio and the pH of the buffer solution. The DAK-GL (DKG) hydrogel matrix is porous, and the freeze-drying step has a three-dimensional and interconnected microstructure in which the pores are due to the formation of ice crystals similar to the structure of other natural macromolecular hydrogels. As shown in the micrographs, the pores in the matrix are about 2–8 μm wide. In addition, a stronger wall appeared due to the ordered aggregates of the polymer chain segments inside the DKG composite hydrogel. The interconnection between the pores can be attributed to the formation of cross-linked networks in the gel. The internal morphology of the cross-linked DKG composite hydrogel depends on the DAK content. Sample 3DKG has the largest pore diameter while the pore diameters of Sample 1DKG and Sample 2DKG are relatively smaller with no significant difference between their internal pore structures. This is mainly because the number of cross-linked nodes between GL and DAK increases as the DAK content increases. The influence of the buffered medium on the DKG matrix morphology is also shown in Figure 4. The 2DKG-A and 2DKG-B have different cross-sectional morphologies and pore diameters after the DKG matrix was completely swelled in the two buffer solutions, with pH being 4.0 and 9.0. As indicated by the comparison with the morphology of the 2DKG hydrogel (relaxed state), the 2DKG-A showed a smaller three-dimensional pore diameter after it swelled in pH 4.0 buffer solution. The 2DKG-B had a larger three-dimensional pore after swelling in pH 9.0 buffer solution.

Starch is another one of the most widely used polysaccharides, KGM is also mixed with starch to prepare some new gels for expanding the applications [45]. KGM-potato starch gels for increasing the stability of carvacrol trapping were successfully prepared by Lafarge et al. [46], to obtain texture features on the gels and a better understanding of their microstructure, Generalized Fourier Descriptors was used to process the SEM images in this study. The results showed that the addition of KGM increased the pore size but prevented the formation of very large pores and therefore reduced the syneresis, but the addition of carvacrol reduced the physical stability of the gel with larger pores and increased syneresis. KGM-rice starch gels were successfully prepared by Charoenrein et al. [47], effects of KGM on the microstructures of complex gels were investigated in this research and the SEM results showed that starch gels with KGM had smaller pores and less well-defined surrounding matrices than those without KGM, meanwhile, meanwhile, KGM could reduce the aggregation of swollen starch granules.

#### 2.3.2. KGM-Protein Compounding

To improve the structure and performance of protein products during the preservation process, KGM and proteins are often compounded to prepare edible gels [21,25,48,49,50]. KGM and egg whites were mixed to prepare gels. The results showed that at 0.06% KGM, the gel strength and water retention capacity were highest. As shown in SEM, the amount of KGM has a significant effect on the structure of the mixed gel. The structure had a two-dimensional and dense network without KGM; with 0.06% KGM, the surface of the gel structure was uneven, with small pore diameters. With 0.10% KGM, there were many pores on the surface of the gel [48].

KGM-wheat gluten mixtures were prepared by Wang et al. [49], KGM showed significant effect on heat-induced changes in gluten protein, this was due to the different driving forces of protein denaturation and different KGM status at various temperatures. Comparing with gluten without KGM, more cataclastic structure included in KGM-gluten mixtures when the heat temperature is 25 °C. However, the network was less compact and the mesh size was bigger at 55 °C, and the layer thickness of samples with KGM increased, this was due to KGM could improve the water-holding capacity of samples by change the conformation and enlarge pores though ice crystal formation.

KGM-*Tilapia* myofibrillar protein (TMP) composite gels were prepared under neutral pH with varying KGM of different molecular characteristics by Jian and his partner [25], γ-irradiation was used to degrade KGM to obtain the degraded KGM with different molecular weight in this study. The results showed that TMP gels contained smooth and compact texture structure but KGM-TMP composite gels existed porous and coarse texture with varying degrees. Moreover, different type of KGM showed significant effect on the size of holes and roughness in morphology of composite gels, the composite gel contained compact and locally smooth morphology when the KGM was degraded by using the irradiation dosage of 100 kGy, it was similar with the morphology in TMP gel except with some fragmentary fine holes on the surface.

#### 2.3.3. KGM-Synthetic Polymer Compounding

**Figure 5:**
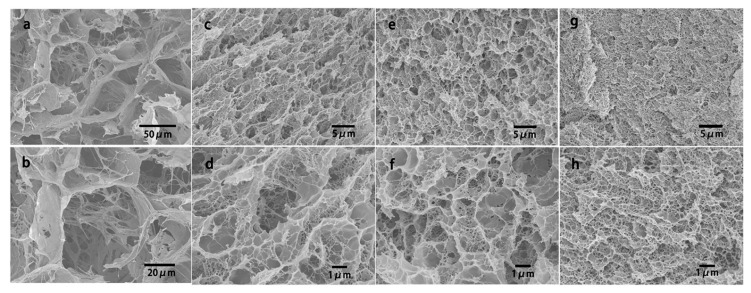
SEM images of a PVA–KGM hydrogel (**a**,**b**) and the PVA–KGM/PAAm DN hydrogels (**c**,**d**: 1 CFT; **e**,**f**: 2 CFT; **g**,**h**: 4 CFT). The AAm concentration used for the preparation of the DN hydrogel is 4 M. CFT is cyclic freeze-thawing times [51].

Li et al. [51] developed a new physical-linked dual-network (DN) hydrogel based on the natural polymer KGM, the synthetic polymer polyvinyl alcohol (PVA) and polyacrylamide (PAAm). The PVA-KGM hydrogel was prepared for the first time using a cyclic freeze-thawing method, and the PVA-KGM/PAAm DN hydrogels were successfully obtained by polymerization. The study was carried out with acrylamide (AAm) content and the cyclic freeze-thawing times as the function of the mechanical strength and microstructure of DN hydrogel. As shown in Figure 5, the PVA–KGM hydrogels contains interconnected porous structure with pore sizes in the range of 40–50 mm, but the DN hydrogels just have pores with sizes in the range of 0.3–5 mm. The PAAm chains lead to the formation of an embedded micro-network in the DN hydrogels. Moreover, the microstructure of the DN hydrogels was also affected by cyclic freeze-thawing times. The pore size of the hydrogel decreases with the increase of the cyclic freeze-thawing times.

**Figure 6:**
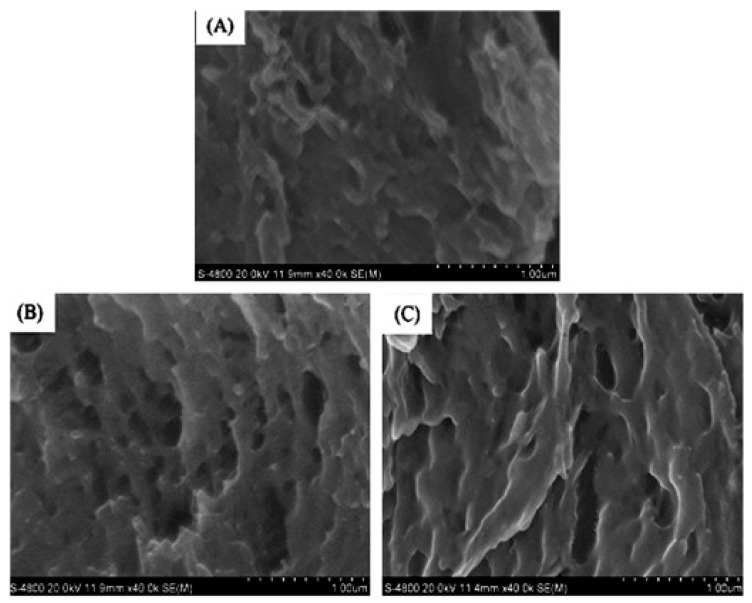
SEM photograph of cross-section of the semi-interpenetrating polymer network (IPN) hydrogels: (**A**) KP07; (**B**) KP08 and (**C**) KP09, the scale bar indicates 100 μm [52].

Liu et al. [52] prepared a new type of pH-sensitive semi-interpenetrating polymer network (IPN) network hydrogel using KGM and poly(aspartic acid) (PAsp) with trisodium trimetaphosphate (STMP) as the cross-linker. They studied how the swelling properties of hydrogel were affected by the component ratio, cross-linking density (STMP concentration), pH, and ionic strength. The structure of semi-IPN hydrogels was characterized with Fourier transform infrared spectroscopy (FT-IR), surface area analysis, and SEM. Based on the SEM images of hydrogel samples, the average pore diameter of the PAsp/KGM semi-IPNs was dependent on the content of STMP. As the content of STMP in the hydrogels increased, the average pore diameter of hydrogels increased, but the further increase of STMP would led the decrease of pore size, this phenomenon was due to the contribution of the cross-linking density becomes predominant. To further investigate the effect of the cross-linker content on the pore structure of the sample, the SEM images of the samples (KP07, KP08 and KP09) were selected and analyzed (Figure 6). The resultHs show that the change in the cross-sectional morphology of semi-IPN hydrogels at different STMP concentrations has the same effect on the average pore diameter of semi-IPN hydrogels as the cross-linker content.

**Figure 7:**
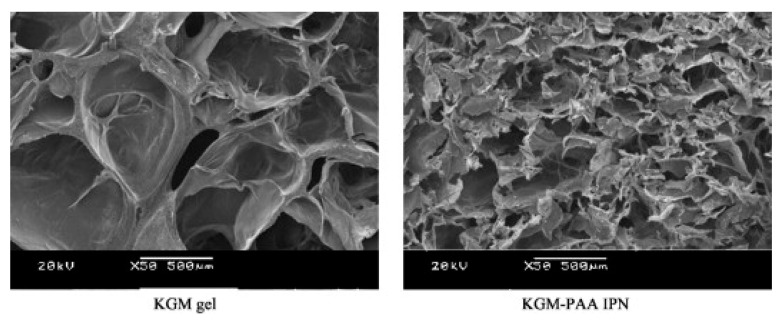
SEM pictures of the cross-section of the freeze-dried KGM hydrogel and KGM/PAA-IPN hydrogel. Magnification 50× [53].

Wen et al. [53] reported the synthesis and properties of IPN network hydrogel system designed for colon-targeted drugs release. The gel consists of KGM and poly(acrylic acid) (PAA) via a cross-linking of *N*,*N*-methylene-bis-(acrylamide) (MBAAm). The cross-sectional morphologies of the freeze-dried KGM hydrogel and KGM/PAA-IPN hydrogel are shown in Figure 7. The KGM hydrogel shows a porous honeycomb structure with many macropores indicating the ability of water to control the amorphous parts of the hydrogel. The pore diameter of KGM/PAA-IPN hydrogels became smaller because acrylic acid entered the pores of the KGM hydrogel and crosslinked with it to form an IPN structure. The average pore diameter of the IPN gel decreases as the crosslink density increases.

### 2.4. Electric Field Preparation

Wang et al. [15] successfully prepared electrochemically reversible KGM-tungsten (T) hydrogels with a direct current (DC) electric field in the presence of sodium tungstate and explored the effects of sodium tungstate concentration, KGM concentration, voltage, and electric processing time on the rheological properties and structure of the gel. The pH experiment showed that the KGM sol containing Na_2_WO_4_·2H_2_O near the positive electrode became acidic, and the negative electrode became alkaline after DC electric fields were applied. Under acidic conditions, WO_4_^2−^ ions were converted to isopolytungstate ions. The Fourier Transform infrared spectroscopy (FT-IR) and Raman studies showed that isopolytungstate ions were adsorbed on the KGM molecular chain and are cross-linked with the –OH group at the C–6 position on the KGM sugar unit. The frequency scanning data show that viscoelastic modulus (i.e., the storage and loss modulus of the gel) increases with increased sodium tungstate concentration, voltage, and electric processing time. The increase in KGM concentration decreases the viscoelastic modulus of the gel. The temperature scanning measurement showed that the resulting gel has high thermal stability. Ultimately, the mechanism of gel formation was proposed, and this work may pave the way for designing and developing KGM gels and polysaccharide gels via DC electric fields.

### 2.5. Cross-Linking of Metal Ions after Modification

Chen et al. [54] developed a photoresponse delivery system composed of gel microspheres made from (2,2,6,6-tetramethylpiperidine-1-oxyl) TEMPO oxidized KGM (OKGM); the COO^–^ group was cross-linked by Fe^3+^ and functional components could be incorporated. The microspheres were degraded by irradiation with (simulated) sunlight to release the embedded components. As demonstrated by proton titration and FT-IR spectroscopy, the oxidation degree (DO) of the OKGM can be well controlled between 15% and 80%. OKGM with DO of 80% was selected to prepare the microspheres because the high COO-content resulted in high density cross-linking of the strong gels. The electrokinetic potential of the OKGM particles increases with pH increases and salt concentration decreases. FT-IR spectroscopy reveals that cross-linking was formed by the two modes coordinated by the COO^−^–Fe^3+^, that is, 68.4% by bridging and 31.6% by single-tooth binding. Thus, the unique properties of the OKGM microspheres enable them to potentially be applied in light-controlled biocompatible delivery systems.

## 3. Application of KGM Gel

### 3.1. Food Industry

#### 3.1.1. Food Additives

Food additives are important for the properties of foods, due to its excellent thickening and gelling properties, KGM has been widely used in food industry. In recent years, KGM has been authorized as a food additive in Europe and classified as GRAS (Generally Recognized as Safe) by the FDA (Food and Drug Administration) [55]. KGM has been used to prepare low fat processed cheese by Felix da Silva, et al. [56], the study indicated that the addition of KGM can improve the rheological and textural properties of low fat processed cheese, meanwhile, enhance its stable behavior. Dai et al. [57] reported that KGM can be used as a fat replacer in the preparation of low-fat and skimmed yogurt, the low-fat and skimmed yogurt with addition of KGM showed stronger and more stable gel structures. Charoenrein et al. [47] reported the effects of KGM as a cryo-gel. Zhou et al. [37] used KGM and low-concentration Na_2_CO_3_ (0.1–0.2% of starch) to prepare KGM-wheat starch gel with a rapid viscosity analyzer. Borreani et al. [58] studied the effects of KGM on the gastric digestion of dairy protein ingredients, they found that the addition of KGM increased the viscosity of dairy protein ingredients during gastric digestion, which probably would increase gastric distention affecting satiety. Li et al. [59] used micronized konjac gel as fat analogue in the preparation of mayonnaise, they found that fat in mayonnaises substituted with konjac gel of not more than 30% was acceptable, and the additions of micronized konjac gel could improve the storage stability of mayonnaise.

KGM gel can also be used as a stabilizer for ice cream to make it taste smooth and delicate; it can also be used as a beer foam stabilizer so that the foam can be small, uniform and can stay on the wall of the glass for a long time after the beer is poured into the glass. As an additive for baking food such as biscuits, cakes, etc. KGM gel enables the product to look smooth and taste light and crisp. It increases the strength and toughness of extra-thin noodles and optimizes the taste. KGM gel can also be used as a clarifying agent and food preservative for fruit juices and alcohol [60]. In the production of beverages with pulp, adding a small amount of konjac powder solution can improve the suspension effect, improve the viscosity of the juice and slurry, adjust the taste, and improve the food quality.

#### 3.1.2. KGM Gel Food

KGM gel foods can be divided into two categories. One is thermally irreversible gel food made from konjac powder. These are typically represented by konjac tofu (cakes, silks) and the derived products such as snow konjac, konjac noodles, konjac slices, and bionic food. The second is thermally reversible gel food such as jelly, pudding, jam, and fat-free soft sweets [60]. KGM-egg white protein gels have been successfully prepared by Li and his partners [48,61] their study showed that KGM could significantly improve the water retention capacity, porous microstructure, hardness, chewiness and springiness, thermal stability and other properties of KGM-egg white protein gel samples. This gel is a self-structuring food with desired sensory and functional properties. Electromyographic has been used to study the effects of mechanical properties and mouthful sizes on natural eating behaviors of two types of soft solid foods. The study showed that konjac mannan-κ-carrageenan-locust bean gum gels with greater fracture strain and work required more mastication effort and less swallowing effort than agar gels with higher elastic modulus [62]. The effects of two different gums (κ-carrageenan or XG) and the xylitol concentration on the textural properties of KGM gels were investigated using response surface methodology by Akesowan [63], The KGM gels optimal conditions were used to product no added sugar, 20% grape juice jelly drinks. The study showed that the KGM jelly drinks containing κ-carrageenan were more acceptable and achieved a higher consumer purchase rate than those containing XG. Noodle is one of staple foods in china, KGM and 0.4% calcium hydroxide had been used to improve the quality of buckwheat noodles by Han et al. [64], the study showed that KGM and 0.4% calcium hydroxide improved the tensile strength and firmness of the cooked noodles, the presence of calcium hydroxide promoted the formation of gel networks in the noodles, and further resulted in better cooking and sensory qualities.

#### 3.1.3. Meat and Fish Industry

In recent years, KGM gel has been widely used in meat and fish industry, such as cryoprotectant, restructured foods, fat replacers, and so on [65,66,67,68,69,70,71,72]. Xiong et al. [50] reported that KGM could be used as a cryoprotectant to mitigate the decrease in salt extractable protein, Ca^2+^-ATPase activity, and total sulphydryl and active sulphydryl contents of myofibrillar protein during frozen storage, meanwhile, to ensure that the other properties of surimi gels were not destroyed, the addition of KGM was suggested at the level of 1%. Moreover, KGM degradation products also showed long time protective effect on frozen grass carp myobrillar [66]. Another article reported that KGM could be used to overcome the weak gelation property of low-quality squid surimi and achieve better gels from them [67]. Liu et al. [68] also used KGM to improve the gel properties of low-quality surimi. Andrés-Bello et al. [69] studied the effect of KGM on some physico-chemical and mechanical properties of restructured gilthead sea bream products, the products without heat-treated contained well water holding capacity and adhesiveness, while the heat-treated products with reduced hardness, cohesiveness and chewiness. In addition, KGM had been used to prepare low-fat restructured pork nuggets by Berry et al. [70]. KGM and seaweed were used to prepare reduced- and low-fat, low-salt (NaCl) frankfurters by Jiménez-Colmenero et al. [71], KGM gel was used as fat replacer in this study and could reduce the fat content over 15% but without noticeable changes in the sensory quality of frankfurters. Furthermore, KGM–starch mixed gels also been used to prepare reduced-fat (~18%) frankfurters, the study showed that KGM-starch mixed gel could be an ideal fat replacer to reduce the fat of frankfurters and enhance its health value [72]. Salcedo-Sandoval et al. [73,74] prepared n-3 PUFA enriched frankfurters by using KGM gel-health oils complexes as fat replacer, this frankfurters was healthier and could be be stored for a long time. Meanwhile, KGM gel also been used in the preparation of low-fat bolognas and sausages [75,76,77].

### 3.2. Drug Carrier

In natural polymers, KGM based gels is often considered a potential carrier of a specific bioactive protein drug delivery system. Xiao et al. [78] incorporated carboxymethyl groups in the KGM structure to reduce the adsorption of water and the solubility of CMKGM. This reduced the molecular hydrophilicity. This modified structure has several promising applications: preparation of biodegradable films [79], enzyme-encapsulated biomaterials [80], and drug carriers [81].

Drug carriers largely determine the action time and utilization of medicines. Traditional medicines are limited by a serious burst release or excessively fast release. More recently, medicine-loaded materials have been prepared using natural polymers and nano materials. KGM can be degraded in the colon but cannot be degraded in the small intestine; thus, it may be used as an adjuvant for colon-targeted drugs. Tablets prepared by wet granulation with KGM/XG/sucrose as the matrix have good mechanical strength and sustained release effect. These are close to grade 0 drug release. At the same time, the drug release effect is affected by the KGM category and the degree of acetylation. Different categories of KGM and different degrees of acetylation lead to different swelling rates and drug release effects of the prepared tablets [82].

In addition to tablet excipients, KGM can also be used to prepare controlled release beads. KGM can be combined with alginate (ALG) and chitosan (CS) to prepare controlled release beads with KGM wrapped within. There are significant dents on the surface of the KGM beads after drug delivery indicating that KGM helps increase the drug payload [83]. The new polymer electrolyte beads can be prepared by electrostatic action in an aqueous medium with CMKGM and CS. These are used for drug delivery systems. Here, beads are sensitive to the pH, and the expansion rate of the beads in the alkaline environment is higher than that in the acidic environment. This results in a relatively good sustained release effect [84]. In addition, the KGM gel can be used for controlled release of the matrix. Under mild conditions, KGM deacetylation and physical cross-linking can be used to prepare a DNA controlled release hydrogel. The DNA release can be controlled by changing the preparation conditions and the structural parameters of the gel [85]. The copolymerization of KGM and acrylic acid and its cross-linking with *N*,*N*-methylene bisacrylamide can form a new type of hydrogel system for colon-targeted drug delivery. The swelling rate of the hydrogel is regulated by the cross-linking degree of the polymer. Cellulase controls hydrogel enzymolysis, and drug release can be controlled by swelling and hydrogel degradation [86].

Wang et al. [26] prepared KGM/SA/GO hydrogels with KGM, GO, and alginate (SA) as raw materials. The in vitro experiments showed that KGM/SA/GO-3 hydrogels can relieve the burst release of 5-fluorouracil (5-FU) and can effectively control the release rate of 5-FU by adjusting the pH.

The KGM microsphere prepared by via oxidative cross-linking [87]. This was used to study the anthocyanin releasing mechanism. Studies have shown that KGM microspheres prepared by oxidative cross-linking can prevent the early release of anthocyanin under gastric conditions and realize persistent release in the intestine. The in vitro release experiment showed that OKGM microspheres can serve as a carrier to deliver biologically active compounds in the intestine.

### 3.3. Tissue Scaffold

KGM has excellent gelation properties and is also non-toxic and biocompatible. KGM hydrogels are widely applied in biomedical fields as a biological material. According to Fan et al. [14], the composite hydrogel can be prepared via a Schiff Base reaction between the aldehyde group of OKGM and the amino group of CMCS, with GO as the nano-additive. The hydrogel scaffolds showed a uniformly interconnected pore structure after freeze-drying. The OKGM/CMCS/GO hydrogel is expected to be an ideal wound dressing because of its moderate water absorption capacity, a compression modulus similar to the soft tissue, and good biocompatibility.

Few studies have addressed the application of KGM-based materials in wound dressings. Recently, by incorporating CS, KGM and KGM composite biomaterials were used as wound dressings. KGM and KGM composite biomaterials can create a moist wound-healing environment that absorbs excess secretions and allows gas exchange. Moreover, they can be easily removed from the wound surface [88].

### 3.4. Absorption Material

As a porous ultra-light material derived from a gel, KGM has an extremely low density, a large surface area, and high mechanical strength. In particular, aerogels are widely used in adsorbing, catalytic, insulating, and sound-proofing materials [89]. According to Chen et al. [90], the network structure of porous KGM nano-microfiber aerogel is constructed by hydrogen bonds in a random and interpenetrating manner. The nano-microfiber structure exists in the KGM aerogel, which is an important reason for its high density and compressive strength. The unique nano-microfiber aerogel can address oil spills by absorbing biodegradable bacteria.

In recent years, hydrogels have also been widely used in the field of adsorbing materials. Most hydrogel materials have a three-dimensional, porous network structure, and their huge surface area is conducive to adsorption. KGM-based hydrogels contain a large number of –OH that can be combined with metal ions and other pollutants through hydrogen bonding. Konjac glucomannan-poly(acrylic acid) hydrogel prepared by KGM and poly(acrylic acid) is an effective adsorbent to adsorb Cu^2+^ from aqueous solution. The adsorption behavior of Cu^2+^ is characterized by the Langmuir isotherm model. Its maximum adsorption capacities at 298, 303, 308, and 313 K are 27.1739, 30.2115, 34.1297, and 41.6667 mg·g^−1^, respectively. The adsorption process is featured with a level-2 model absorption [2]. According to Gan et al. [91], KGM and GO can be combined to prepare a KGM/GO hydrogel by means of Ca(OH)_2_ as the cross-linking agent, which performs better than a pure KGM hydrogel in absorbing methyl blue and methyl orange. The KGM/GO has great potential as a sewage adsorbent. 

Wu et al. [17] prepared a new type of CMKGM-immobilized microsphere adsorbent with a sol-gel method to absorb fluoride ions in water. The adsorption nicely matches a Langmuir isotherm model. As indicated by absorption kinetics and thermodynamics studies, this new material can significantly adsorb the fluoride ions in water; thus, it can be used as a promising adsorbent for fluoride removal.

### 3.5. Sensing Material

Yang et al. [92] demonstrated that biomass-derived carbonaceous aerogels can achieve excellent properties by being hierarchically structured in an architecture similar to that of honeycomb. Hence, the research team used KGM and flexible silica nanofibers to prepare super elastic carbon nanofiber aerogels (CNFAs) similar to the honeycomb that has an ordered structure. Due to the synergetic effect between the ordered, porous fiber structure and the fully bonded carbon nanofiber, CNFAs have an extremely low density, very good cyclic compressibility, zero Poisson’s ratio, excellent thermal stability, elastic response to conductivity, and high-pressure sensitivity. Being able to detect dynamic pressure across a wide range with high sensitivity means that this aerogel can realize real-time in situ monitoring of pressure signals like the human body blood pulse.

## 4. Conclusions

In conclusion, the gelation performance makes KGM unique and superior to other natural macromolecules. Therefore, there is great potential to study the microstructure and application of KGM gel materials in gel products, pharmaceutical carriers, tissue scaffolds, and adsorption materials. The most recent preparation methods of KGM gel emphasize freeze-thawing, borate cross-linking, polymer compounding, electric field preparation, and cross-linking of metal ion after modification. The microstructures were studied via SEM.

As a polymer material, KGM is safe and non-toxic. Thus, it is a “new favorite” in the food, pharmaceutical, and chemical industries. As a new type of functional material, it deserves in-depth research and development and has promising application prospects. It currently has a huge development space in natural polymer polysaccharides. For example, KGM can be combined with microfluidic spinning to achieve drug delivery, wound healing, tissue engineering and regenerative medicine. As a drug release carrier, KGM has outstanding advantages such as low price, safety and non-toxicity. This will not lead to burst release during drug release. Although KGM nano-gel controlled-release materials have received much attention, further studies are necessary to understand its release mechanism.
